# Direct functional consequences of ZRS enhancer mutation combine with secondary long range SHH signalling effects to cause preaxial polydactyly

**DOI:** 10.1016/j.ydbio.2014.05.025

**Published:** 2014-08-15

**Authors:** Edward J. Johnson, David M. Neely, Ian C. Dunn, Megan G. Davey

**Affiliations:** a Division of Developmental Biology, The Roslin Institute and R(D)SVS, University of Edinburgh, Easter Bush, Midlothian EH25 9RG, UK; b Division of Genetics and Genomics, The Roslin Institute and R(D)SVS, University of Edinburgh, Easter Bush, Midlothian EH25 9RG, UK

**Keywords:** Sonic hedgehog, ZRS, Polydactyly, HOXA13, Cyclopamine, SAG

## Abstract

Sonic hedgehog (SHH) plays a central role in patterning numerous embryonic tissues including, classically, the developing limb bud where it controls digit number and identity. This study utilises the polydactylous *Silkie* (*Slk*) chicken breed, which carries a mutation in the long range limb-specific regulatory element of *SHH*, the ZRS. Using allele specific *SHH* expression analysis combined with quantitative protein analysis, we measure allele specific changes in *SHH* mRNA and concentration of SHH protein over time. This confirms that the *Slk* ZRS enhancer mutation causes increased *SHH* expression in the posterior leg mesenchyme. Secondary consequences of this increased SHH signalling include increased FGF pathway signalling and growth as predicted by the SHH/GREM1/FGF feedback loop and the Growth/Morphogen models. Manipulation of Hedgehog, FGF signalling and growth demonstrate that anterior-ectopic expression of *SHH* and induction of preaxial polydactyly is induced secondary to increased SHH signalling and Hedgehog-dependent growth directed from the posterior limb. We predict that increased long range SHH signalling acts in combination with changes in activation of *SHH* transcription from the *Slk* ZRS allele. Through analysis of the temporal dynamics of anterior *SHH* induction we predict a gene regulatory network which may contribute to activation of anterior *SHH* expression from the *Slk* ZRS.

## Highlights

• Overexpression of posterior *SHH* in the limb bud can cause preaxial polydactyly.
• Increased activation of SHH/GREM/FGF feedback and growth induces *Slk* preaxial polydactyly.
• Autoregulated expression of *SHH* can occur within 1.5–2 h in the limb bud.

## Graphical abstract

**Figure:**
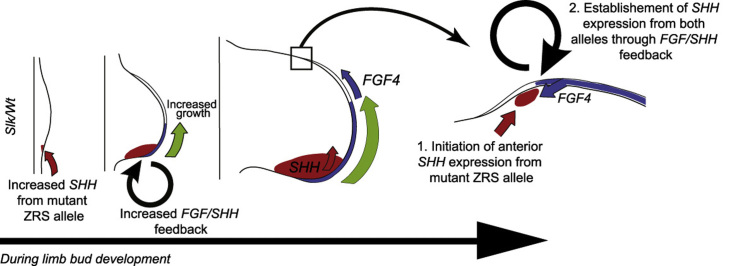

## Introduction

The zone of polarising activity (ZPA) is a transient area of posterior limb bud mesenchyme with the ability to induce and pattern extra digits when grafted to the anterior border of a host wing (Saunders and Gasseling, 1968). The ZPA was one of the first examples of an ‘organiser’ tissue, having the predicted morphogen-like capability of patterning the three digits of the chick wing in a time and concentration dependent manner (Tickle et al., 1975; Smith, 1980). First discovered in the chick, it is now recognised that all patterned vertebrate limbs use a ZPA mechanism to determine a specific number and identity of digits. Since then the limb bud has been the focus of intense experimentation and modelling with the aim of understanding the “universal mechanism whereby the translation of genetic information into spatial patterns of differentiation is achieved” (Wolpert, 1969).

Molecular studies have elucidated many components of this ‘universal mechanism’. The ZPA morphogen is now established as Sonic hedgehog (SHH), which is expressed in, and mediates the action of the ZPA in a time and concentration dependent manner (Smith, 1980; Tickle, 1981; Riddle et al., 1993; Yang et al., 1997; reviewed Tickle and Barker 2012). SHH co-ordinates limb growth and digit patterning simultaneously by maintaining Hedgehog-dependent growth from the posterior limb during the early digit patterning phase, resulting in digit pattern that is regulated by concentration and length of exposure to SHH directly and by the expansion of the limb field at later stages of limb development (the Growth/Morphogen model; (Harfe et al., 2004; Towers et al., 2008; Zhu et al., 2008)). This is mediated through a positive feedback loop with Fibroblast Growth Factors (FGFs) expressed in the overlying ectoderm, mediated by mesenchymal BMP-antagonist *Gremlin1*, promoting outgrowth of the limb (The SHH/GREM1/FGF feedback loop; Laufer et al., 1994; Niswander et al., 1994; Lewandoski et al., 2000; Michos et al., 2004; Bénazet et al., 2009; Galli et al., 2010; Zeller, 2010). Both *SHH* expression and limb out-growth is terminated when high levels of FGF signalling inhibits *GREM1* expression which disrupts the SHH/GREM1/FGF feedback loop (Verheyden and Sun, 2008).

The localisation, timing of *SHH* expression and strength of SHH signalling is tightly controlled to create a localised morphogen source, key to creating a signalling gradient in order to specify digit identity (Wolpert, 1969). The regulation of *SHH* expression is crucial for correct digit patterning. In the posterior limb, *SHH* has been shown to be autoregulative in a negative manner as exposure to high concentrations of SHH protein induces cell death of *SHH* expressing cells (Sanz-Ezquerro and Tickle, 2000) while conversely inhibition of Hedgehog signalling can increase *Shh* expression (Scherz et al., 2007). In addition, implantation of *SHH*-expressing cells in the anterior of the limb can induce *SHH* in endogenous tissue after 48 h (Duprez et al., 1999) demonstrating that as in the neural tube, the anterior of the limb bud has the potential to express *SHH* (Tanaka et al., 2000) in response to SHH signalling, although the time lag suggests that this is likely to be indirect. Native *SHH* autoregulation in the developing limb bud, in un-manipulated circumstances has yet to be reported.

*SHH* expression is restricted to precise anatomical locations in the lung, larynx, pharynx, gut and limb by a number of highly conserved long range, tissue-specific, *cis*-regulatory elements (Lettice et al., 2003, Sagai et al., 2005, 2009). The limb specific enhancer is known as the ZPA Regulatory Sequence (ZRS; (Lettice et al., 2003); also MFCS1; (Sagai et al., 2005)). Mutations within the ZRS are associated with preaxial (anterior) polydactyly in multiple species and are thought to drive ectopic expression of *SHH* in the anterior portion of the limb bud, acting as a *de facto* ZPA. (Lettice et al., 2003, 2008; Park et al., 2008; Dunn et al., 2011). It has been proposed that *cis*-regulatory regions contain multiple binding sites for essential transcription factors (homotypic clustering; (Gotea et al., 2002)). This has been demonstrated in the ZRS, which contains multiple ETS factor binding sites with both repressive and activating effects on *SHH* expression in the limb, which when disrupted by mutations within the ZRS, cause polydactyly in humans (Lettice et al., 2012).

Previously we mapped the dominant chicken *Polydactyly* locus (*Po*) in the *Silkie* (*Slk*) chicken breed, which has anterior (preaxial) polydactyly in the leg, to a novel single nucleotide polymorphism (SNP) in the chicken ZRS (Dunn et al., 2011). Chicken feet normally have four digits, labelled anterior–posterior from I to IV. Preaxial polydactyly in the *Slk* breed is most commonly observed as an extra digit II (II,I,II,III,IV). Unlike other ZRS mutants, the *Slk* ZRS SNP is not within nor creates a predicted ETS binding site. Uniquely among ZRS mutants, induction of polydactyly in the *Slk* leg is both time and posterior ZPA dependent. This suggests that ectopic anterior *SHH* expression is the consequence of intact limb bud gene expression and signalling feedback loops which are abnormally activated by aberrant posterior gene expression. Indeed, we have shown that *FGF4* and *GREM1* are expressed ectopically in the *Slk* leg (Dunn et al., 2011). Tissue recombination experiments, however, demonstrate that induction of *Slk* polydactyly is genotype specific, as ectopic *SHH* is not induced in anterior *Wt* tissue recombined with *Slk* posterior leg mesenchyme (Dunn et al., 2011). Based on these observations we have previously proposed a model, based on the Growth/Morphogen model (Towers et al., 2008) which suggests that extra SHH signalling observed in the posterior *Slk* leg may cause growth and long-range patterning effects which leads to preaxial polydactyly (Dunn et al., 2011). To test this hypothesis we propose that induction of anterior *SHH* and preaxial polydactyly in the *Slk* is dependent on three conditions which we test here; an increase in SHH protein from posterior mesenchyme, upregulation of normal leg responses to increased SHH signalling, such as growth and additional FGF signalling, and additional activity of the ZRS conferred by the *Slk* ZRS SNP in both anterior and posterior tissue. Based on our evidence we propose a model to explain the temporal regulation of polydactyly and ectopic *SHH* expression by the *Slk* ZRS.

## Materials and methods

### Animal maintenance

Polydactylous (*Slk*) and White Leghorn (*WL*) and *talpid*^*3*^ (*ta*^*3*^) chicken lines are maintained at the Roslin Institute under UK Home Office licence after ethical review. Birds were genotyped from gDNA using primers for ZRS SNP and the *SHH* promoter non-synonymous SNP as per Dunn et al. (2011). For breeding purposes and to control for breed specific traits, all experiments were undertaken using embryos produced by a *Slk*^*Po*^*/Slk*^*Wt*^×*WL*^*Wt*^*/WL*^*Wt*^ cross. For simplification, unless otherwise stated resulting embryos will be referred to in the text as the following: *Slk*^*Po*^*/WL*^*Wt*^=*Slk/Wt*, *Slk*^*Wt*^*/WL*^*Wt*^=*Wt/Wt*

### Embryo manipulations

Tungsten foil was inserted into small slits prepared between somites 29 and 30 in the leg mesenchyme using fine tungsten needles. Cyclopamine (Sigma) was prepared to a 1 µg µl^−1^ concentration in 45% 2-hydropropyl-β-cyclodextrin (Sigma) and smoothened agonist (SAG, Calbiochem) was prepared to a concentration of 0.2 µg µl^−1^ concentration in water. 5 µg of cyclopamine or 1 µg SAG was injected directly onto the embryo (so that the entire embryo was surrounded with compound), via a small hole made in the vitelline membranes. AG1-X2 beads were soaked in 1 mg ml^−1^ all-trans retinoic acid (Sigma), 10 mM SU5402 (Sigma) or 1 mg ml^−1^ trichostatin A (Sigma) for 20 min, control beads in DMSO. After washes with DMEM beads were inserted into stage 18–20HH limbs using fine tungsten needles. E10 embryos were stained with alcian green and cleared with methyl salicylate. Digit identity in the foot was assigned by phalanx number and numbered I, II, III, IV from anterior–posterior. Numbering of wing digits is 1, 2, 3 from anterior to posterior. Nile Blue staining was performed as per Dunn et al. (2011). Total limb bud area and ANZ area (post Nile Blue staining) was determined using the “Perimeter” function of Image J, with area calculated in-program.

### Protein quantification

Stage 21HH and 24HH legs were homogenised in RIPA buffer (Fisher) containing protease inhibitors, centrifuged, and the supernatant is collected. Protein concentration was estimated using a DC Protein Assay kit (Bio-Rad). Recombinant mouse SHH N-terminus protein (R&D Systems) was used as a positive control. Protein samples were loaded as individual limbs per lane. Proteins were separated by electrophoresis using pre-cast 12% gels (Invitrogen), transferred to nitrocellulose membranes by standard procedures. Membranes were blocked in Odyssey Blocking Buffer (Licor), incubated with 1:100 rabbit anti-SHH H-160 (Santa Cruz, sc9024) 1:2500 mouse anti-γ-tubulin (Sigma, T5326) 4 °C overnight, followed by goat anti-rabbit 680CW (Licor, 926-32221) goat anti-mouse 800CW (Licor, 926-32210) for 1 hour. Membranes were dried and signal detected using an Odyssey Infrared Imager (Licor). Bands were quantified using Image Studio software, and normalised to γ-tubulin protein.

### Quantitative real-time PCR and RFLP assays

Fertilised Silkie/White Leghorn eggs were incubated at 38 °C, windowed and staged (Hamburger and Hamilton, 1951) and dissected between 17 and 27HH. Wings were taken whole and legs either whole (stages 17–23HH) or dissected into posterior and anterior halves (stages 24–27HH). Tissue dissociation, cDNA synthesis, RFLP–PCR and densitometry were carried out as per Dunn et al. (2011). *SHH* primers: Forward CCCACCTGCTCTTTGTGG; and reverse AGGAGCCGTGAGTACCAATG. qRT-PCR was carried out using a Brilliant III Ultra-Fast SYBR Green QPCR mix (Agilent) in a Stratagene MX 3000. Standard curves of known molar concentration of PCR product were prepared in triplicate from leg cDNA. Absolute quantities of *SHH* and *LBR* were calculated using standard curves generated by MX software (Stratagene). Primers: *SHH* Forward: CCCCAAATTACAACCCTGAC Reverse: CATTCAGCTTGTCCTTGCAG; *LBR* Forward: GGTGTGGGTTCCATTTGTCTACA Reverse: CTGCAACCGGCCAAGAAA.

### Whole-mount *in situ* hybridisation

RNA probe synthesis and whole-mount RNA *in situ* hybridisation were performed as per Nieto et al. (1996). Probes were synthesised from the following templates: *SHH* (Roelink et al., 1994), *HOXA13* (Nelson et al., 1996), *HOXD13* (ChEST414K15, Ark Genomics), and *PTCH1* (Marigo et al., 1996).

### Bioinformatics

Predicted transcription factor binding sites were determined *in silico* using MatInspector (Genomatix, (Cartharius et al., 2005)) databases, and further analyses were performed using GEISHA (Bell et al., 2004) and compared to microarray data generated by the eChickAtlas (Wong et al., 2013).

### Electrophoretic mobility shift assays

5′ biotin-labelled oligonucleotides (Sigma) were annealed to produce double-stranded DNA probes. Nuclear extracts were prepared from anterior and posterior halves of stage 24HH *Slk/WL* legs using the NE-PER Nuclear and Cytoplasmic Extraction kit (Thermo Scientific). 3 µg of nuclear extract was incubated with binding buffer (20 mM HEPES, pH 7.5, 50 mM KCl, 1 mM DTT, 0.5 mM MgCl_2_), 1 µg poly dI.dC (Thermo Scientific) and un-labelled competitor (if required) for 10 min at room temperature. 10 fmol biotin-labelled probe was added, and incubated for further 20 min. Reactions were separated by native electrophoresis at 100 V, 4 °C, using equilibrated 6% polyacrymalide gels and 0.5× TBE. Nucleotides were transferred onto positively charged nylon membranes, developed using a Chemiluminescent Nucleic Acid Detection module (Thermo Scientific) and detected using autoradiography film. Oligos:

*Wt* ZRS 5′–3′AATGAGCTTTCATTGCATGCTTTCATTATT;
*Wt* ZRS 3′–5′ AATAATGAAAGCATGCAATGAAAGCTCATT;
*Slk* ZRS 5′–3′ AATGAGCTTTAATTGCATGCTTTCATTATT;
*Slk* ZRS 3′–5′ AATAATGAAAGCATGCAATTAAAGCTCATT.

## Results

### Increased SHH in the posterior *Slk* leg bud causes expression of anterior ectopic *SHH* and preaxial polydactyly

**Fig. 1:**
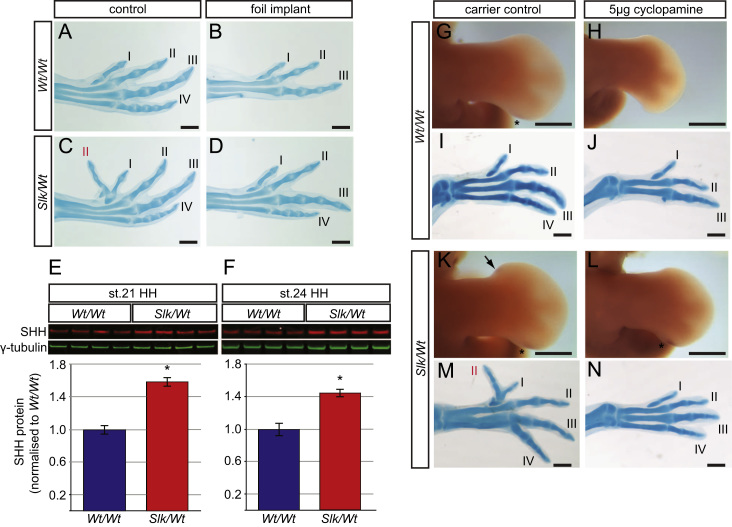
Increased SHH protein from the posterior limb is required for the formation of preaxial polydactyly. (A–D) Foil insertion between the anterior and posterior leg bud causes loss of posterior digits (B) and prevents preaxial polydactyly (D *n*=3/3). (E,F) Increased SHH protein in stage 21 (E) and 24HH (F) *Slk/Wt* legs (±SEM, *n*=4, *P*<0.0005). (G–N) Cyclopamine causes loss of posterior and anterior *SHH* (H, *n*=3/3, L, *n*=5/5) compared to controls (G, K; arrows and asterisks) and causes loss of posterior digits (J, *n*=7/14) and preaxial ectopic digits (N, *n*=13/16) compared to controls (I, M). Scale=0.5 mm.

The anterior *Slk* leg develops an ectopic area of *SHH* expression at late stage 25HH, which leads to preaxial polydactyly (Arisawa et al., 2006; Dunn et al., 2011). Localised surgical ablation has previously demonstrated that ectopic anterior *SHH* expression and preaxial polydactyly in the *Slk* leg requires posterior *Slk* leg bud mesenchyme (Dunn et al., 2011). To demonstrate that the prevention of polydactyly in these manipulations was due to the loss of a diffusible factor contained in the posterior tissue, rather than the ablated tissue itself we interrupted communication between the posterior and anterior mesenchyme using foil barriers inserted into stage 20HH leg buds. Control manipulations in which developing leg buds were cut but no foil barrier was inserted, caused no change in digit number (Fig. 1A, *Wt/Wt* digit pattern I,II,III,IV; Fig. 1C, *Slk/Wt* digit pattern II,I,II,III,IV). Insertion of a foil barrier between anterior and posterior leg bud mesenchyme, however, disrupted posterior digit patterning in both *Wt/Wt* and *Slk/Wt* legs (Fig. 1B, *n*=5/6) and in addition *Slk/Wt* legs failed to form ectopic anterior digits (Fig. 1D, *n*=3). By preventing communication between posterior and anterior leg mesenchyme, ectopic anterior *SHH* expression and preaxial polydactyly was not induced in *Slk/Wt* leg buds (Fig. S1A, Fig. 1D). This demonstrates that *Slk/Wt* posterior leg tissue contains a diffusible inductive signal which induces ectopic anterior *SHH* and preaxial polydactyly.

The *Slk* ZRS SNP alters posterior *SHH* expression and causes upregulation of *GLI1*, a gene responsive to Hedgehog signalling (Dunn et al., 2011). Thus we expect that SHH is the posteriorly localised, diffusible factor, mediating induction of preaxial polydactyly in the *Slk* leg. Quantitative Western blot analysis confirmed that SHH protein levels were significantly increased in posterior *Slk/Wt* leg buds at stages 21 and 24HH compared to *Wt/Wt* leg buds (Fig. 1E, 1.6-fold increase in SHH protein in *Slk/Wt* legs stage 21HH; Fig. 1F, 1.4-fold increase in SHH protein in *Slk/Wt* legs stage 24HH. *n*=4 for each stage, *P*>0.0005). To confirm that the posterior factor required for the induction of anterior *SHH* and preaxial polydactyly was SHH, we inhibited SHH signalling at stages 17–20HH (prior to expression of ectopic anterior *SHH*) using cyclopamine (Chen et al., 2002a). Of the treated embryos, 7/14 *Wt/Wt* embryos lost digit 4 (Fig. 1J), while 13/16 *Slk/Wt* embryos maintained four digits but failed to develop ectopic digits (Fig. 1I). The remaining 3/16 legs were polydactylous. As the majority of the *Slk/Wt* legs did not form anterior-ectopic digits, this confirms that anterior *Slk* polydactyly is dependent on posterior *SHH* expression. To test if anterior *SHH* expression was lost in *Slk/Wt* legs we repeated the experiment by treating embryos with cyclopamine or carrier solution at stage 17HH, and assayed *SHH* expression at stage 25HH via whole mount *in situ* hybridisation. Whilst the carrier solution control had no effect (Fig. 1G,K), cyclopamine inhibited *SHH* expression in the posterior of *Wt/Wt* leg (Fig. 1H, *n*=3/3). *SHH* expression was absent in the anterior *Slk/Wt* leg whilst posterior *SHH* was maintained (Fig. 1L; *n*=5/5). This demonstrates that SHH signalling is required to induce anterior ectopic *SHH* expression in *Slk/Wt* legs and that there is a reduced ability of cyclopamine to downregulate posterior *SHH* expression or digit IV induction in *Slk/Wt* legs compared to *Wt/Wt* legs. We conclude that overexpression of *SHH* in the ZPA of the posterior leg bud is the primary inductive signal which induces anterior *SHH* expression and preaxial polydactyly in the anterior *Slk* leg.

### Increased activation of SHH/GREM/FGF feedback network and growth, controls induction of *Slk* preaxial polydactyly

**Fig. 2:**
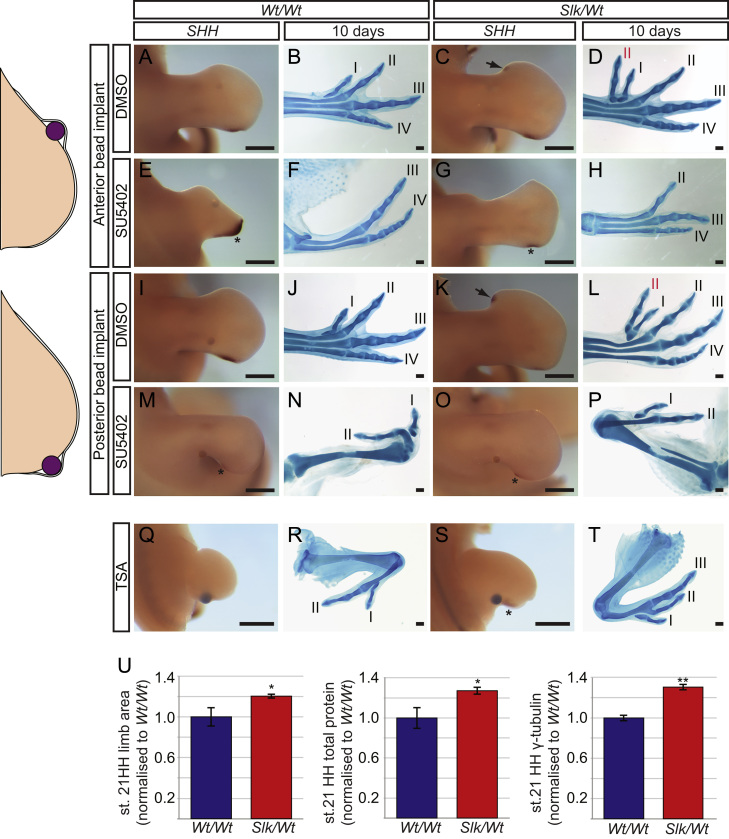
Inhibition of FGF signalling and growth prevent polydactyly. (A–P) Localised inhibition of FGF signalling at stage 20HH prevents ectopic *SHH* and polydactyly. Insertion of DMSO soaked beads into the anterior (A–D) and posterior (I–L) AER-mesenchyme did not alter *SHH* expression or digit pattern for each genotype (A,I, arrows in C, K). SU5402 beads placed in the anterior AER-mesenchyme caused loss of digits I and II in both genotypes (F,H, *n*=4/4), including preaxial digits in *Slk/Wt* (H, *n*=3/3). Ectopic *SHH* was prevented in *Slk/Wt* (G, 2/2) whereas posterior *SHH* remained in both genotypes (E,G; asterisks, *n*=4/4). SU5402 administered to the posterior AER-mesenchyme caused a reduction in ZPA *SHH* (M,O; asterisks, *n*=5/6) and prevented anterior ectopic *SHH* in *Slk/Wt* (**O***n*=2/2). Digits III and IV were lost from each genotype (N,P, *n*=9/11) as well as preaxial digits in the *Slk/Wt* (P, *n*=4/5). (Q–T) TSA applied to the posterior AER-mesenchyme ablated *SHH* expression in *Wt/Wt* legs (Q) and caused loss of posterior digits (R, *n*=9/11), whilst *Slk/Wt* embryos retained *SHH* expression (S) and retained greater numbers of posterior digits, but lost ectopic digits (T, *n*=11/15) when compared with DMSO controls (I–L). (U) Increased leg area, total protein (*Wt/Wt n*=6, *Slk/Wt n*=18, *p*<0.05,±SEM) and γ-tubulin protein (*n*=4, *p*<0.00005,±SEM) in *Slk/Wt* legs. Scale=0.5 mm.

The inability of cyclopamine to fully repress posterior *SHH* and digit IV identity in *Slk/Wt* legs suggests that the SHH/GREM1/FGF feedback loop is not fully disrupted by cyclopamine treatment. In addition, *Slk* legs exhibit prolonged *SHH* expression and extended *FGF4* and *Gremlin* domains (Dunn et al., 2011) which may be responsible for the induction of anterior ectopic *SHH*. To test this possibility we inhibited FGFR signalling by inserting beads soaked in SU5402 (Mohammadi et al., 1997) between the AER and mesenchyme at stage 20HH, prior to the induction of preaxial polydactyly. Application of SU5402 between the AER and mesenchyme of the anterior limb caused a localised loss of tissue, resulting in the loss of digits I and II (Fig. 2F *n*=4/4), including preaxial polydactylous digits (Fig. 2H *n*=4/4). Digits III and IV were unaffected. Anterior expression of *SHH* was not observed in *Slk/Wt* leg buds (Fig. 2G *n*=2/2), even though posterior *SHH* expression was maintained in all leg buds (Fig. 2E,G *n*=4/4). It is likely that SU5402 treatment in the anterior mesenchyme prevents a response to ectopic *SHH* by inhibiting cell proliferation, leading to a loss of preaxial digits. We then extended this analysis by applying SU5402 to the posterior limb bud. Digits III and IV were lost in both *Wt/Wt* and *Slk/Wt* (Fig. 2N,P, *n*=9/11). Posterior *SHH* expression was weakly maintained in both *Wt/Wt* and *Slk/Wt* legs (Fig. 2M,O, asterisks, *n*=5/6). *Slk/Wt* legs failed to develop preaxial polydactyly (Fig. 2P, *n*=4/5) or express anterior *SHH* (Fig. 2O, *n*=2/2). The loss of tissue growth and *SHH/FGF* feedback in the posterior limb affected not only digits IV and III but also prevented the formation of preaxial digits in the anterior limb, whilst leaving digits I and II unaffected. These findings demonstrate that inhibition of posterior FGFR can prevent anterior ectopic *SHH* and subsequent preaxial polydactyly by reducing posterior *SHH* expression and tissue growth.

SHH signalling controls and integrates both proliferation and patterning in the limb (Growth/Morphogen model; Towers et al., 2008; Zhu et al., 2008). To identify whether additional SHH from the posterior leg mesenchyme causes increased limb growth in *Slk/Wt* leg buds, we measured leg bud area and protein content in *Slk/Wt* leg bud at stage 21HH. Leg bud area, total protein content and γ-tubulin were all increased in *Slk/Wt* compared to *Wt/Wt* (Fig. 2U). To establish if increased SHH-dependent limb bud expansion is required for the induction of anterior-ectopic *SHH* and preaxial polydactyly in *Slk/Wt* leg buds, we locally inhibited growth by implanting TSA-soaked beads into the posterior leg bud mesenchyme, proximal to the ZPA, at stage 18–19HH (Towers et al., 2008; Zhao et al., 2009; Towers et al., 2011). Unlike previous observations, where application of TSA at stage 20HH inhibits anterior digit formation while maintaining posterior digit identity (Towers et al., 2008), *Wt/Wt* TSA-treated legs either lost posterior digits or had posterior–anterior digit identity transformation (Fig. 2R *n*=9/11). *Slk/Wt* TSA-treated legs had posterior–anterior digit identity transformation but only one leg bud had a loss of digit IV (Fig. 2T *n*=13/15). Furthermore anterior-ectopic digits failed to form in 11/15 *Slk/Wt* TSA-treated legs (Fig. 2T). As is seen in Towers et al. (2008) (24 h after treatment with TSA) *SHH* expression was lost in *Wt/Wt* legs (Fig. 2Q), but only reduced in *Slk/Wt* legs (arrows, Fig. 2S). This correlates with the increased number of digits retained in *Slk/Wt* leg, which were also of a more posterior SHH dependent nature (digit III, although digit IV was not observed). We attribute our posterior digit loss to the timing of TSA application, during the early phase of SHH-dependent digit patterning. SU5402 and TSA treatments both exhibited the same prevention of anterior *SHH* and preaxial digit formation, suggesting that the effect of SU5402 was due to a localised loss of growth. However in accordance with the Growth/Morphogen model, induction of preaxial polydactyly was dependent on limb bud expansion driven by growth in the posterior leg.

### *Slk* wings are more responsive to inductive signals activating *SHH* expression

**Fig. 3:**
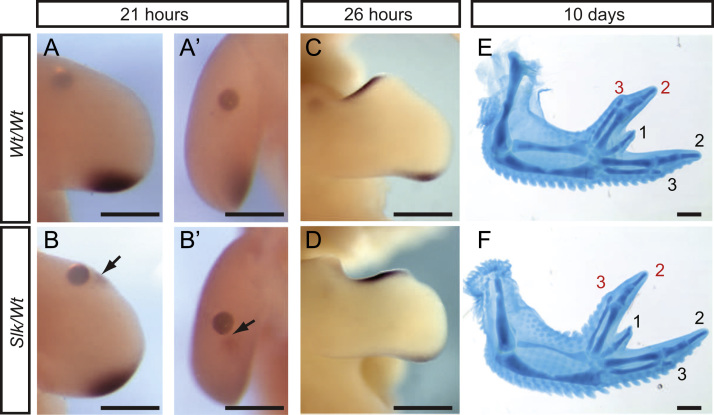
Retinoic acid treatment induces *SHH* earlier in *Slk* wings Implantation of RA beads to the anterior AER-mesenchyme for 21 h induced ectopic *SHH* in the *Slk/Wt* wing (arrows, B,B׳, *n*=6), but not the *Wt/Wt* (A,A׳, *n*=5). Exposure for 26 h induced ectopic *SHH* in both *Wt/Wt* and *Slk/Wt* wings (C,D, *Wt/Wt n*=8, *Slk/Wt n*=5). Day 10 digit pattern identical for both genotypes (additional digits indicated in red; E,F). Scale=0.5 mm.

**Fig. 4:**
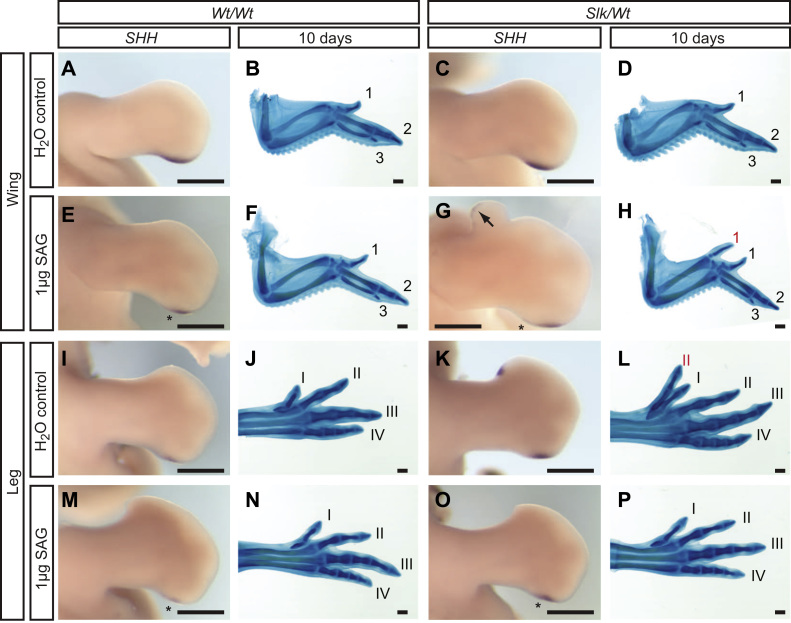
Hyperactivation of the Hedgehog pathway prevents polydactyly. (A–H) *SHH* expression and digit pattern in wings treated with water (A–D) or SAG (E–H). SAG did not induce ectopic *SHH* expression (C) or affect digit pattern (D) in *Wt/Wt* wings. Ectopic *SHH* (G, arrow; *n*=3/5) and additional preaxial digits (H; *n*=4) formed upon SAG treatment in *Slk/Wt* wings. (I–P) *SHH* expression and digit pattern in legs treated with water (I–L) or SAG (M–P). SAG did not induce ectopic *SHH* expression (M) or affect digit pattern (N) in *Wt/Wt* legs. SAG treatment prevented anterior ectopic *SHH* expression (O; *n*=5) and preaxial polydactyly in *Slk/Wt* legs (P; *n*=9/12). Scale=0.5 mm.

Ectopic anterior *SHH* and leg preaxial polydactyly in *Slk/Wt* legs, therefore, is primarily dependent on increased SHH produced by the posterior mesenchyme. Recombination experiments between *Wt* and *Slk* leg tissue, however, have shown that posterior mesenchyme is not entirely sufficient to induce polydactyly (Dunn et al., 2011). Although allelic imbalance is also observed in the wing ZPA, and therefore the *Slk* ZRS SNP is not leg specific, preaxial polydactyly and ectopic anterior *SHH* expression is only observed in the leg in *Slk* birds and not the wing (Arisawa et al., 2006). We utilised the lack of ectopic *SHH* in the anterior *Slk* wing to investigate the *SHH* transcription response in *Slk/Wt* to retinoic acid (RA) which induces expression of *SHH* in *Wt* wings after implantation of a bead soaked in 1 mg ml^−1^ RA after 24 h (Riddle et al., 1993). Following implantation of RA-soaked beads in to the anterior wings of *Wt/Wt* and *Slk/Wt* embryos, *Wt/Wt* wings did not express *SHH* after 21 h (Fig. 3A,A′, *n*=5) whereas a small area *SHH* expression was detected distal to the bead in *Slk/Wt* wings (arrow, Fig. 3B,B′, *n*=6). Wings of both genotypes showed ectopic expression of *SHH* after 26 h of incubation (Fig. 3C,D; *Wt/Wt n*=8, *Slk/Wt n*=5), and both genotypes form identical mirror image digit duplications (Fig. 3E,F). To further examine the ability of *Slk* tissue to initiate *SHH* expression we utilised a Smoothened agonist (SAG) to activate the Hedgehog pathway in the *Slk/Wt* wing (Frank-Kamenetsky et al., 2002; Chen et al., 2002b). Titration of SAG activity in *Wt/Wt* wings found that 5 µg SAG/embryo at 17–20HH induced an additional digit 2 (not shown), whereas treatment with 1 µg SAG/embryo did not induce *SHH* expression (Fig. 4A,E,I,M *n*=3/3) or polydactyly (Fig. 4B,F,J,N *n*=6/6) in the wing or leg. Application of 1 µg SAG/embryo to *Slk/Wt* embryos, however, induced ectopic anterior *SHH* expression (arrow, Fig. 4G *n*=3/5) and polydactyly in *Slk/Wt* wings (Fig. 4H *n*=4/12). Surprisingly application of 1 µg SAG/embryo prevented ectopic *SHH* and *PTCH1* expression and polydactyly in the *Slk/Wt* leg (Fig. 4O,P *n*=9/12; Fig. S2 *n*=1). The ability of the *Slk/Wt* wing to express *SHH* in response to RA earlier than *Wt/Wt* and at sub-optimal concentrations of SAG confirms that the *Slk* ZRS can also act in the wing if the correct conditions are provided. It has previously been shown that expression of *SHH* is controlled in the wing by apoptosis in an area of posterior mesenchyme known as the posterior necrotic zone (Sanz-Ezquerro and Tickle, 2000; Zuzarte-Luís and Hurlé, 2002) and it has been proposed that different patterns of apoptosis between the wing and leg may underlie the limb specific ability to autoregulate SHH levels (Dunn et al., 2011). Analysis of cell death in the anterior necrotic zone (ANZ) in the wing and leg by Nile blue staining shows that the ANZ area is significantly reduced in the *Slk/Wt* leg compared to *Wt/Wt* (Fig. S3C,D). The *Slk/Wt* wing also exhibits reduced ANZ area (Fig. S3A,B). However, comparing ANZ area of the *Slk/Wt* wing and leg shows that whilst cell death is reduced, the wing still maintains robust cell death compared to the anterior leg (Fig. S3E). Therefore we propose that a lack of anterior *SHH* expression in the *Slk* wing may be a consequence of maintained apoptotic cell death.

### *SHH* controlled by *Slk* ZRS is expressed before *SHH* controlled by the *Wt* ZRS

**Fig. 5:**
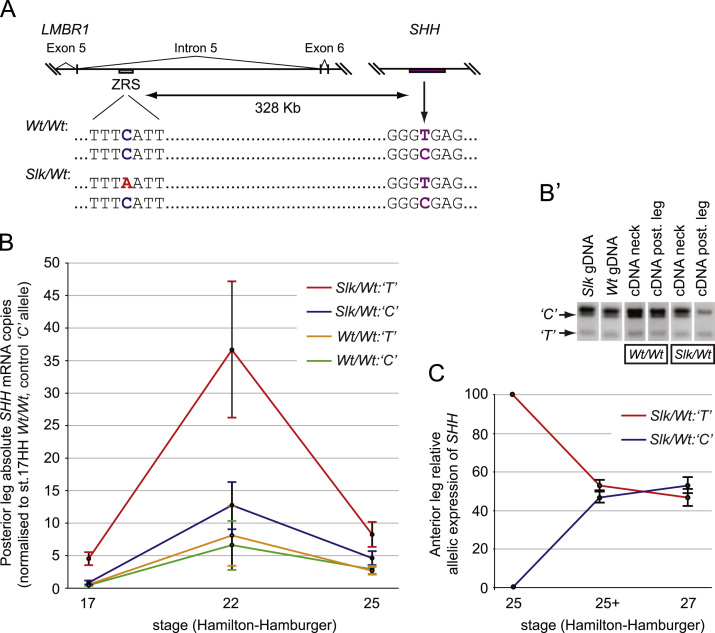
Absolute and relative quantification of allelic contribution to *SHH* expression in posterior and anterior legs. (A) The *Slk* C>A SNP (red) is located in the 794 bp ZRS region residing within intron 5 of the *LMBR1*gene, 328Kb upstream of the *SHH* gene in chicken chromosome 2 (Dorshorst et al., 2010). A non-synonymous SNP within the *SHH* promoter (C>T, arrow) was utilised to track allelic expression. (B) Combined data from absolute quantitation of *SHH* mRNA and relative expression from each allele in stages 17, 22 and 25HH posterior legs. In *Wt/Wt* posterior legs, *SHH* is expressed equally from both alleles (orange and green series). In *Slk/Wt* posterior legs the majority of *SHH* expression arise from the mutant *Slk/Wt:‘T’* allele (red series) compared to the wild type *Slk/Wt:‘C’* allele (blue series) (*n*=5 for each stage). (B′) Example of RFLP assay to determine relative allelic expression of *SHH*. Genomic DNA, neck cDNA and *Wt/Wt* posterior leg cDNA exhibit equal allelic expression, whilst *Slk/Wt* posterior leg cDNA shows reduced contribution from the wild type allele. (C) Onset of ectopic *SHH* expression occurs completely from the mutant *Slk* allele at stage 25HH (red series), with both alleles contributing equally from late stage 25HH onwards.

We then utilised the *Slk/Wt* leg to elucidate the dynamics of *SHH* autoregulation when SHH signalling is perturbed. To do this we constructed an allelic-specific expression profile of *SHH* in the posterior leg utilising a non-synonymous SNP within the *SHH* gene (Fig. 5A). This allowed us to examine expression of the *Wt SHH* allele in an abnormal SHH signalling environment (*Slk/Wt* leg buds). We carried out semi-quantitative RFLP assays on leg buds from stages 17 to 27HH to assess relative expression from *Wt* or *Slk SHH* alleles (Fig. 5B′, Fig. S1B). As expected there was equal expression from both alleles in *Wt/Wt* leg buds from 17 to 27HH (Fig. S1B, green and yellow series). In contrast at stage 17HH, *SHH* in the *Slk/Wt* leg was largely expressed from the mutant *Slk* allele (~85%) which continued to account for ~65% of *SHH* expression throughout leg development (Fig. S1B, red series). As the RFLP assay is restricted to determining relative contribution of each allele, we performed quantitative RT-PCR analysis (qRT-PCR) in *Wt/Wt* and *Slk/Wt* legs to determine *SHH* mRNA at stages 17, 22 and 25HH. The general trend in both genotypes was an increase in *SHH* expression between stage 17HH and 22HH followed by a decrease between stage 22HH and −25HH (Fig. 5B). Overall *SHH* expression in *Slk/Wt* legs was significantly increased at all three stages compared to the control *Wt/Wt* legs. By combining the RFLP data with qRT-PCR data and normalising to stage 17HH *Wt/Wt SHH* expression, we were able to accurately compare expression of each allele in *Slk/Wt* relative to *Wt/Wt* legs. In *Slk/Wt* legs at all stages, the *Slk SHH* allele is expressed at increased levels (Fig. 5B, approximately 3 fold increase at stage 22HH red series) while the *Wt SHH* allele level is comparable to *Wt SHH* alleles in *Wt/Wt* legs (Fig. 5B; compare blue series to yellow and green series). Analysis of relative *SHH* allele contribution in the anterior *Slk/Wt* leg, where expression is initiated at stage 25HH, confirmed that ectopic expression initially occurs only from the *Slk SHH* allele (Fig. 5C, red series), followed within half a stage (1.5–2 h) by expression from the *Wt SHH* allele (Fig. 5C, blue series). This suggests that once ectopic expression is initiated from the mutant *Slk SHH* allele, the *Wt SHH* allele contributes in an autoregulative manner in order to maintain ectopic *SHH* expression.

Cyclopamine has previously been shown to increase *SHH* expression in the ZPA (Scherz et al., 2007). We sought to characterise allelic contribution to this increase in ZPA *SHH* upon inhibition of the Hedgehog pathway. Cyclopamine treatment at stage 17HH normalised allelic imbalance in *Slk/Wt* legs at stage 19HH, with equal expression from each allele (Fig. S1C). qRT-PCR and ISH of cyclopamine treated *Slk/Wt* legs showed a significant increase of *SHH* expression (Fig. S1D), suggesting that inhibition of the Hedgehog pathway drives increased *SHH* expression from each allele, regardless of genotype. Both the 1.5–2 h transcription response of the *Wt SHH* allele during ectopic *SHH* and the increased posterior *SHH* induced by cyclopamine (~6 h) suggest that *SHH* transcription can be rapidly induced via autoregulation.

### *Slk* ZRS SNP causes changes in transcription factor binding

**Fig. 6:**
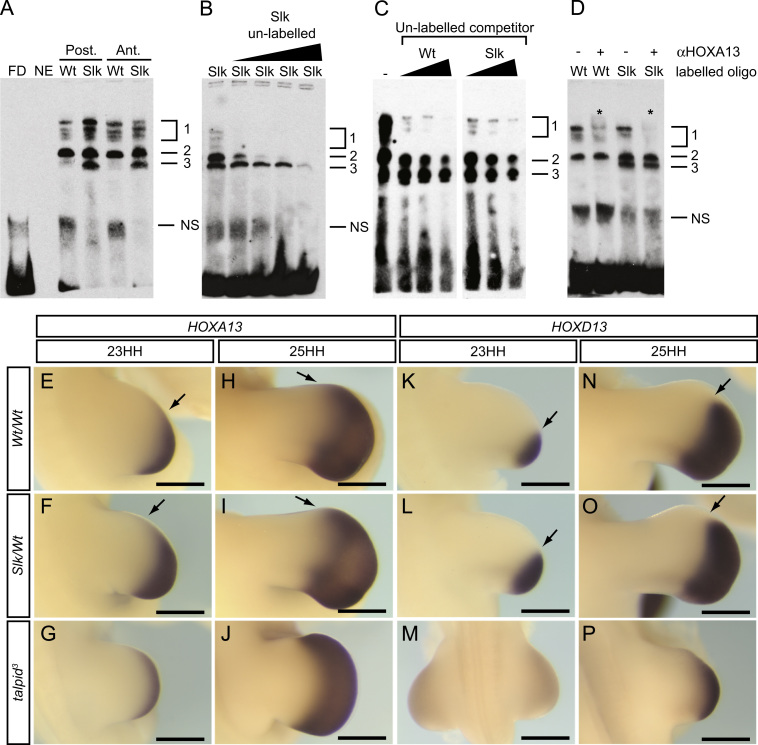
Protein binding profiles to *Wt* and *Slk* ZRS, and gene expression patterns of possible ZRS-interacting proteins. (A) Binding profiles of nuclear proteins to *Wt* and *Slk* ZRS DNA. Exclusive binding of Band 3 to *Slk* DNA. Nuclear proteins from posterior and anterior leg buds exhibit identical binding profiles. (B) Un-labelled *Slk* DNA (100×, 200×, 400×, 2000×) outcompete Bands 1–3. (C) Competition assays with un-labelled *Wt* and *Slk* DNA against labelled 10 fmol *Slk* DNA (5×, 10×, 20×). *Wt* DNA competes Bands 1 and 2, whereas Band 3 is still present. (D) Incubation with anti-HOXA13 causes supershift of upper bands (Band 1) in both *Wt* and *Slk* DNA. (E–J) *HOXA13* expression extends distally towards the anterior leg during limb development. Extended anterior expression in *Slk/Wt* (F,I, arrows) compared to *Wt/Wt* legs (E,H, arrows). (K–P) *HOXD13* expression follows a similar pattern, but does not extend as far towards the anterior at stage 25HH (N,O, arrows) although the anterior border of expression is extended in *Slk/Wt* (compare *Wt/Wt* K,N arrows to *Slk/Wt* L,O, arrows). *talpid*^*3*^ legs exhibits uniform extended *HOXA13*, *HOXD13* expression (G,J,M,P). FD; free DNA control (no nuclear protein), NE; nuclear extract control (no labelled probe), NS; non-specific. Scale=0.5 mm.

The ZRS is a long-range enhancer of *SHH*, containing many transcription factor binding sites which mediate *SHH* expression through a combination of repression and activation (Lettice et al., 2012). To assess if the *Slk* ZRS mutation either creates or disrupts a transcription factor binding site, we examined the binding profiles of the *Wt* and *Slk* ZRS sequences surrounding the SNP site by EMSA. Labelled *Wt* and *Slk* ZRS probes yielded similar binding profiles when incubated with *Slk*/*Wt* leg nuclear extract, including a grouped set of upper bands (Band 1) and Band 2 (Fig. 6A). In addition the labelled *Slk* probe yielded an additional ‘Band 3’ (Fig. 6A). Nuclear extract from posterior and anterior halves of leg buds produced identical binding patterns (Fig. 6A). Whereas competing *Slk* labelled probe with unlabelled *Slk* probe depleted all three bands at varying concentrations (Fig. 6B), unlabelled *Wt* probe only competed Bands 1 and 2, and Band 3 remained (Fig. 6C). This suggests that Band 3 is due to a genuine protein:DNA interaction caused by an alteration of protein binding by *Slk* ZRS SNP, whereas Band 2 may represent a protein binding elsewhere on the probe, uninvolved with the *Slk* SNP site.

In order to identify candidate transcription factors which may account for the additional *Slk* ZRS SNP specific Band 3 we identified a number of conditions that must be met by a candidate transcription factor in order to bind to the *Slk* ZRS SNP. Candidate transcription factors must be co-expressed in areas of *Slk* limbs which express *SHH* and which exhibit allelic imbalance; the posterior regions of the wing and leg during stages 17–27HH and the proximal-anterior mesenchyme of the *Slk* leg from stage 25HH. Candidate factors may bind to the *Slk* ZRS in the anterior *Slk* wing, but ectopic *SHH* is prevented due a lack of cell death reduction (Fig. S3B). In addition the candidate transcription factor would have *SHH* dependent expression, as ectopic anterior *SHH* in the leg is induced by increased SHH signalling from posterior mesenchyme, the *Slk* wing expresses ectopic *SHH* in response to SAG (Fig. 4G), and anterior *SHH* expression is orevented by application of cyclopamine (Fig. 1L). The *Slk* ZRS SNP is a *C*>A change in a highly conserved region of the ZRS, which creates a small AT-rich region with similarity to canonical HOX binding sites (Georges et al., 2010; Hueber and Lohmann, 2008; Knosp et al., 2007). We used MatInspector to search for possible binding sites for transcription factors created by the *Slk* ZRS SNP. This suggested the creation of 19 potential transcription factor binding sites, of which 13 contained homeodomains (Table S1; Genomatix; Cartharius et al., 2005). While analysis of published and publically available gene expression patterns (GEISHA, Bell et al., 2004; eChickAtlas, Wong et al., 2013) with Affymetrix microarray expression analysis *Wt* and *talpid*^*3*^ chicken limbs (Bangs et al., 2010) determined that most MatInspector candidates did not fulfil the candidate gene criteria (Table S1), *HOXA13*, however, had appropriate spatiotemporal expression in *Wt* limbs. We therefore compared *HOXA13* with *HOXD13* in *Slk/Wt*, *Wt/Wt* embryos to confirm that it fulfilled our candidate criteria, as well as in *talpid*^*3*^ embryos to confirm its responsiveness to SHH signalling. We used *HOXD13* as a comparison a it has been shown to bind to the *Wt* ZRS in complex with HAND2 (Galli et al., 2010) and has previously been shown not to be expressed in the *Slk* anterior leg until after initiation of *SHH* (Dunn et al., 2011). We confirmed that *HOXA13* expression domain is anteriorly expanded in the *Slk/Wt* at stage 23HH (Fig. 6, compare E to F), the crucial time point for induction of preaxial polydactyly in the *Slk* leg (Dunn et al., 2011). Although *HOXA13* is expressed in the anterior of both *Slk/Wt* and *Wt/Wt* legs at 25HH (Fig. 6, compare H to I), we have shown that at this point the *Slk/Wt* limb is refractory to manipulation of polydactyly at this point and therefore the action of a candidate geen must act prior to this. Expanded expression of *HOXA13* in *talpid*^*3*^ legs at all stages (Fig. 6G,J) demonstrates that it is a SHH-responsive gene (due to the loss of Gli repressor function in *talpid*^*3*^ limbs; (Davey et al., 2006)). In comparison *HOXD13* expression is also expanded anteriorly across the *Slk/Wt* legs at both stage 23HH and stage 25HH compared to *Wt/Wt* (Fig. 6 compare K to L and N to O) although the anterior border of expression does not reach the domain of ectopic *SHH* expression (Fig. 6O). As *HOXA13* fulfilled the criteria we had determined to bind ectopically to the *Slk* ZRS, we performed supershift EMSA using an anti-HOXA13 antibody to test if the *Slk* specific ‘Band 3’ was due to ectopic HOXA13 binding. This, however, shifted the upper bands (Fig. 6D, Band 1), not Band 3, suggesting that although HOXA13 interacts with both the *Wt* and *Slk* ZRS *in vitro*, it does not account for the *Slk* specific Band 3. In conclusion, HOXA13 is not responsible for ectopic *SHH* induction in the anterior *Slk* leg. The nature of the *Slk* exclusive Band 3 (Fig. 6A–D, Band 3), however, remains unknown.

## Discussion

### *Slk* preaxial polydactyly is dependent on direct misexpression of *SHH* and on secondary long range patterning events

In this study we investigate how the novel *Slk* ZRS SNP controls expression of ectopic *SHH*. In a previous analysis of the *Slk* leg we proposed a model, based on the Growth/Morphogen model (Towers et al., 2008) which suggested that the increased SHH signalling observed in the posterior *Slk* limb causes both growth and patterning effects which underlie the induction of preaxial polydactyly (Dunn et al., 2011). To test this model we examined three conditions; the expression of *SHH* and concentration of SHH protein sourced from the posterior mesenchyme, the subsequent changes in the limb regulatory network, and the action of the *Slk* ZRS in controlling *SHH* expression compared to *Wt* ZRS. Here we have shown these conditions form the basis of the induction of preaxial polydactyly and increased/ectopic *SHH* expression in the *Slk* leg, giving an insight into the dynamics and long-range patterning effects which can be induced in response to perturbed SHH signalling. In addition, our analysis has yielded important data on the autoregulation of *SHH* transcription in limb development, when SHH signalling is altered. These findings have relevance for all areas – normal and pathological – in which *SHH* is expressed. Small molecule inhibitors of Hedgehog signalling, such as Vismodegib, which has a similar mode of action to cyclopamine, have been used successfully in the treatment of basal cell carcinomas in which Hedgehog signalling is activated by mutations in *SMO* or *PTCH1*, downstream of expression of a Hedgehog ligand (Von Hoff et al., 2009). The action of Vismodegib on other tumours which are dependent on excess expression of Hedgehog ligand, such as pancreatic adenocarcinoma, has not been so successful (Sarris et al., 2013). Out work suggests that in response to a loss of Hedgehog signalling through small molecule inhibition, expression of the Hedgehog ligand can be highly and rapidly upregulated (Fig. S1C, D), which may subsequently cause an increase in Hedgehog signalling once the dose of Hedgehog inhibitor has lost efficacy.

Previously we had shown that the *Slk* ZRS causes a number of changes in *SHH* expression; *SHH* is expressed both in a larger posterior domain and for a longer period. We also demonstrated, indirectly, that the formation of preaxial polydactyly is dependent on posterior leg tissue (Dunn et al., 2011). In this study we have shown that more SHH is produced by posterior cells, and importantly, that there is a proportional increase in SHH protein to leg volume (Fig. 1E,F). Inhibition of Hedgehog signalling in the posterior leg (Fig. 1G–N) confirmed that the dependence of preaxial polydactyly induction is due to increased SHH protein originating in posterior leg mesenchyme.

The final requirement of our model was that alterations in *SHH* and growth in the leg must still be able act upon anterior tissue of the correct genotype. This was based on recombination experiments which demonstrated that additional SHH produced in the posterior *Slk* mesenchyme alone was not sufficient to induce preaxial polydactyly (Dunn et al., 2011). Perturbation of SHH signalling via application of RA or SAG to the wing bud suggests that the *Slk* ZRS SNP increases tissue responsiveness to exogenous treatment, causing endogenous tissue to express *SHH* at an earlier time point than the *Wt* equivalent (Fig. 3B,B′, Fig. 4G). Thus we propose that in *Slk* legs additional signalling from the posterior mesenchyme, which is not sufficient to induce *SHH* expression in *Wt* anterior leg tissue, is sufficient in *Slk* anterior mesenchyme. Surprisingly application of SAG inhibited preaxial polydactyly formation in the *Slk* leg (Fig. 4P). SAG application has been shown to upregulate *Ptch1* in mouse embryos (Frank-Kamenetsky et al., 2002) which we believe would change the diffusion dynamics of the additional SHH protein in the posterior *Slk* leg bud, limiting its action to the posterior mesenchyme. SAG is also known to be an inhibitor of Hedgehog signalling if added in excess or in combination with high levels of endogenous Hedgehog signalling (Frank-Kamenetsky et al., 2002).Therefore the posterior *Slk* leg may be more likely to reach the SAG concentration threshold for Hedgehog pathway inhibition than the *Wt* leg.

The striking absence of preaxial digits in the *Slk* wing may be explained by the environment of anterior wing tissue. Recent work has shown that temporal differences in the onset of ANZ formation, coupled with AER regression from the presumptive anterior digit forming mesenchyme, might be responsible for the reduced number of digits in the chicken wing (Nomura et al., 2014). We had previously observed reduced cell death in the anterior *Slk* leg (Dunn et al., 2011). The retention of significant ANZ size in the wing compared to the highly reduced ANZ of the *Slk* leg (Fig. S3) could explain why ectopic *SHH* and preaxial digits are never observed in the *Slk* wing. The requirement of anterior AER-mesenchyme interaction to induce *SHH* in the anterior *Slk* leg (Fig. 2G,H) may not be achieved in the *Slk* wing due to the time-dependent regression of the AER away from proximal-anterior mesenchyme. Our data suggests that even with increased SHH-dependent growth in posterior tissue, anterior tissue must be responsive (reduced ANZ cell death and necessary genotype) in order to form preaxial digits.

We have demonstrated that the *Slk* ZRS exhibits a different binding profiles compared to the *Wt* ZRS (Fig. 6), although the identity of the binding complex exclusive to the *Slk* ZRS (Fig. 6A, Band 3) remains unknown. HOXA13 binds both the *Wt* and *Slk* ZRS *in vitro*, and does not appear to be responsible for the additional band (Band 3) binding to the *Slk* ZRS. However, a caveat in our approach is that the *in vitro* binding affinity of HOX proteins may not represent the greater specificity of *in vivo* DNA:HOX protein interactions (Georges et al., 2010). We are further investigating the binding dynamics at this locus.

**Fig. 7:**
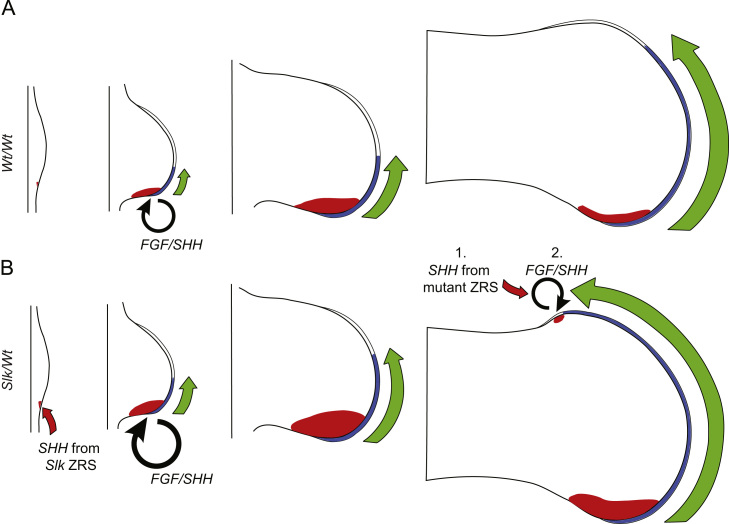
Model depicting the dependence of the mutated ZRS and FGF/SHH feedback loop on ectopic *SHH* expression. (A) Expression of *SHH* (red), and *FGF4* (blue) in *Wt* legs, along with regions of Hedgehog-dependent growth (green arrows) over time. *FGF/SHH* feedback loops form upon formation of ZPA and AER. (B) Presence of *Slk* ZRS SNP increases *SHH* at stage 17 (red arrow). Continued increase in posterior *SHH* expression increases Hedgehog-dependent growth compared to *Wt* (larger green arrows). Increased *SHH* drives increased *FGF4* expression. At stage 25HH ectopic *FGF4* reaches the anterior limb where ectopic *SHH* initiates from the mutant *Slk* ZRS allele, establishing a *de novo*, ectopic *FGF/SHH* feedback loop.

The time-dependent element to the induction of anterior preaxial polydactyly by posterior *SHH* expressing tissue, exemplified by the delay between posterior *SHH* expression at stage 17HH and the expression of *SHH* in the anterior limb at late stage 25HH (Fig. 7), is not observed in other polydactylous models such as the AUS and AC human mutations (Lettice et al., 2012). In AUS and AC ZRS mutants a combination of *ETS* genes are expressed throughout the mouse limb in a manner that is neither SHH, nor time dependent (Lettice et al., 2012; Ristevski et al., 2002) and therefore activation of the transcription of *SHH* in the anterior of the limb bud is not time dependent as it is in the *Slk* ZRS mutant. This highlights that even closely associated SNPs may have widely varying mechanisms by which their action is precipitated.

### Dynamics of autocrine regulation of *SHH* expression in the limb

Our work suggests that mutations within the ZRS, combined with ectopic growth and increased feedback loops cause preaxial polydactyly (Fig. 7A,B). *SHH* expression is upregulated in *Slk/Wt* posterior legs from stage 17HH (Fig. 5B). Subsequent increase in SHH protein causes an increase in Hedgehog-dependent leg growth (Fig. 7, green arrows) and feeds into the SHH/FGF/GREM feedback loop by driving increased *FGF4* expression (Fig. 7B; Dunn et al., 2011). By stage 25HH, ectopic *FGF4* in the AER is expressed in the anterior *Slk* leg (Dunn et al., 2011). At this point ectopic *SHH* expression is initiated, mediated by altered transcription factor binding to the *Slk* SNP site (Fig. 6A, Band 3). Initial expression is driven solely from the *Slk* ZRS (Fig. 5C) and triggers a *de novo*, ectopic SHH/FGF/GREM feedback loop. Autoregulation of *SHH* expression occurs, with both alleles contributing to the ectopic *SHH* that results in preaxial polydactyly. The temporal and inductive aspects of this study propose a novel model for the mechanics and timing of preaxial polydactyly induction. As an example of a common human developmental disorder in a highly conserved regulatory element it illustrates how closely related mutations can have diverse outcomes in the developmental biology underpinning the phenotype.

## Contributions

EJJ undertook experimental work, analysis and manuscript preparation. DMN contributed cyclopamine manipulations. ICD developed experimental approaches. MGD conceived and supervised the study and undertook manuscript preparation and intellectual contribution.

## Appendix A Supporting information

Fig. S1 Effect of foil barrier insertion on anterior *SHH*, allelic profile of ZPA *SHH* expression in *Wt/Wt* and *Slk/Wt* legs, and the effect of cyclopamine on *SHH* expression.(A) Insertion of a foil barrier interrupting the posterior–anterior axis prevents ectopic *SHH* forming in the anterior of *Slk/Wt* leg buds, whilst the control still maintains anterior *SHH* (arrow). (B) A semi-quantitative profile of allelic contribution to *SHH* expression in the posterior leg was generated by RFLP assays across limb development (*n*≥3 for each stage and genotype, ±SEM). *Wt/Wt* limbs show equal expression of each allele from stage 17HH–25HH (green and orange series). *Slk/Wt* limbs exhibit *SHH* expression mostly from the *Slk/Wt:‘T’* allele (red series), with contribution from the *Slk/Wt:‘C’* allele eventually becoming similar to the *Wt/Wt* limb (blue series). (C) Addition of cyclopamine at stage 17HH normalised allelic contribution by stage 19HH (purple and brown series). (D) *SHH* expression was increased in *Slk/Wt* legs at stage 19HH upon cyclopamine treatment at stage 17HH as shown by *in situ* hybridisation and qPCR (bars±SEM). Scale=0.5 mm.

Fig. S2 *PTCH1* expression in SAG-treated *Wt/Wt* and *Slk/Wt* wings and legs SAG treatment did not induce ectopic *PTCH-1* in *Wt/Wt* wings (**A,C**) or legs (E,G), but increased growth was observed in the treated leg (G, *n*=4). In *Slk/Wt*, SAG treatment induced ectopic *PTCH-1* in wings (B,D; arrow, *n*=1), yet prevent endogenous ectopic *PTCH-1* in *Slk/Wt* legs (F,H, *n*=1). Scale=0.5 mm.

Fig. S3 Differences in ANZ cell death may explain lack of preaxial digits in the *Slk* wing Cell death in the ANZ is reduced in both the wings (A,B) and legs (C,D) of *Slk/Wt* embryos. Quantitation of ANZ area **(**E, bars=SEM**)** shows significant reduction in *Slk/Wt* limbs; with a greater reduction in ANZ area in the *Slk/Wt* leg compared to the *Slk/Wt* wing. (*Wt/Wt n*=6, *Slk/Wt n*=16). Scale=0.125 mm.

## References

[bib1] Arisawa K., Yazawa S., Atsumi Y., Kagami H., Ono T. (2006). Skeletal analysis and characterization of gene expression related to pattern formation in developing limbs of Japanese Silkie Fowl. J. Poult. Sci..

[bib2] Bangs F., Welten M., Davey M.G., Fisher M., Yin Y., Downie H., Paton B., Baldock R., Burt D.W., Tickle C. (2010). Identification of genes downstream of the Shh signalling in the developing chick wing and syn-expressed with Hoxd13 using microarray and 3D computational analysis. Mech. Dev..

[bib3] Bell G.W., Yatskievych T.A., Antin P.B. (2004). GEISHA, a whole-mount in situ hybridization gene expression screen in chicken embryos. Dev. Dyn..

[bib4] Bénazet J.-D., Bischofberger M., Tiecke E., Gonçalves A., Martin J.F., Zuniga A., Naef F., Zeller R. (2009). A self-regulatory system of interlinked signaling feedback loops controls mouse limb patterning. Science.

[bib5] Cartharius K., Frech K., Grote K., Klocke B., Haltmeier M., Klingenhoff A., Frisch M., Bayerlein M., Werner T. (2005). MatInspector and beyond: promoter analysis based on transcription factor binding sites. Bioinformatics.

[bib6] Chen J.K., Taipale J., Cooper M.K., Beachy P.A. (2002). Inhibition of Hedgehog signaling by direct binding of cyclopamine to smoothened. Genes Dev..

[bib7] Chen J.K., Taipale J., Young K.E., Maiti T., Beachy P.A. (2002). Small molecule modulation of smoothened activity. Proc. Natl. Acad. Sci. USA.

[bib8] Davey M.G., Paton I.R., Yin Y., Schmidt M., Bangs F.K., Morrice D.R., Smith T.G., Buxton P., Stamataki D., Tanaka M. (2006). The chicken talpid3 gene encodes a novel protein essential for Hedgehog signaling. Genes Dev..

[bib9] Dorshorst B., Okimoto R., Ashwell C. (2010). Genomic regions associated with dermal hyperpigmentation, polydactyly and other morphological traits in the Silkie chicken. J. Hered..

[bib10] Dunn I.C., Paton I.R., Clelland A.K., Sebastian S., Johnson E.J., McTeir L., Windsor D., Sherman A., Sang H., Burt D.W. (2011). The chicken polydactyly (Po) locus causes allelic imbalance and ectopic expression of Shh during limb development. Dev. Dyn..

[bib11] Duprez D., Lapointe F., Edom-Vovard F., Kostakopoulou K., Robson L. (1999). Sonic hedgehog (SHH) specifies muscle pattern at tissue and cellular chick level, in the chick limb bud. Mech. Dev..

[bib12] Frank-Kamenetsky M., Zhang X.M., Bottega S., Wichterle H., Dudek H., Bumcrot D., Wang F.Y., Jones S., Shulok J., Rubin L.L. (2002). Small-molecule modulators of Hedgehog signaling: identification and characterization of smoothened agonists and antagonists. J. Biol..

[bib13] Galli A., Robay D., Osterwalder M., Bao X., Bénazet J.-D., Tariq M., Paro R., Mackem S., Zeller R. (2010). Distinct roles of Hand2 in initiating polarity and posterior Shh expression during the onset of mouse limb bud development. PLoS Genet..

[bib14] Georges A.B., Benayoun B.A., Caburet S., Veitia R.A. (2010). Generic binding sites, generic DNA-binding domains: where does specific promoter recognition come from?. FASEB J..

[bib15] Gotea V., Visel A., Westlund J.M., Nobrega M.A., Pennacchio L.A., Ovcharenko I. (2002). Homotypic clusters of transcription factor binding sites are a key component of human promoters and enhancers. Genome Res..

[bib16] Hamburger V., Hamilton H.L. (1951). A series of normal stages in the development of the chick embryo. J. Morphol..

[bib17] Harfe B.D., Scherz P.J., Nissim S., Tian H., McMahon A.P., Tabin C.J. (2004). Evidence for an expansion-based temporal Shh gradient in specifying vertebrate digit identities. Cell.

[bib18] Hueber S.D., Lohmann I. (2008). Shaping segments: Hox gene function in the genomic age. BioEssays.

[bib19] Knosp W.M., Saneyoshi C., Shou S., Bächinger H.P., Stadler H.S. (2007). Elucidation, quantitative refinement, and *in vivo* utilization of the HOXA13 DNA binding site. J. Biol. Chem..

[bib20] Laufer E., Nelson C.E., Johnson R.L., Morgan B.A., Tabin C. (1994). Sonic hedgehog and Fgf-4 act through a signaling cascade and feedback loop to integrate growth and patterning of the developing limb bud. Cell.

[bib21] Lettice L.A., Heaney S.J.H., Purdie L.A., Li L., De Beer P., Oostra B.A., Goode D., Elgar G., Hill R.E., De Graaff E. (2003). A long-range Shh enhancer regulates expression in the developing limb and fin and is associated with preaxial polydactyly. Hum. Mol. Genet..

[bib22] Lettice L.A., Hill A.E., Devenney P.S., Hill R.E. (2008). Point mutations in a distant Sonic hedgehog cis-regulator generate a variable regulatory output responsible for preaxial polydactyly. Hum. Mol. Genet..

[bib23] Lettice L.A., Williamson I., Wiltshire J.H., Peluso S., Devenney P.S., Hill A.E., Essafi A., Hagman J., Mort R., Grimes G. (2012). Opposing functions of the ETS factor family define Shh spatial expression in limb buds and underlie polydactyly. Dev. Cell.

[bib24] Lewandoski M., Sun X., Martin G.R. (2000). Fgf8 signalling from the AER is essential for normal limb development. Nat. Genet..

[bib25] Marigo V., Scott M.P., Johnson R.L., Goodrich L.V., Tabin C.J. (1996). Conservation in hedgehog signaling: induction of a chicken patched homolog by Sonic hedgehog in the developing limb. Development.

[bib26] Michos O., Panman L., Vintersten K., Beier K., Zeller R., Zuniga A. (2004). Gremlin-mediated BMP antagonism induces the epithelial–mesenchymal feedback signaling controlling metanephric kidney and limb organogenesis. Development.

[bib27] Mohammadi M., McMahon G., Sun L., Tang C., Hirth P., Yeh B.K., Hubbard S.R., Schlessinger J. (1997). Structures of the tyrosine kinase domain of fibroblast growth factor receptor in complex with inhibitors. Science.

[bib28] Nelson C.E., Morgan B.A., Burke A.C., Laufer E., DiMambro E., Murtaugh L.C., Gonzales E., Tessarollo L., Parada L.F., Tabin C. (1996). Analysis of Hox gene expression in the chick limb bud. Development.

[bib29] Nieto M.A., Ketan P., Wilkinson D.G. (1996). in situ hybridization analysis of chick embryos in whole mount and tissue sections. Methods Cell Biol..

[bib30] Niswander L., Jeffrey S., Martin G.R., Tickle C. (1994). A positive feedback loop coordinates growth and patterning in the vertebrate limb. Nature.

[bib31] Nomura N., Yokoyama H., Tamura K. (2014). Altered developmental events in the anterior region of the chick forelimb give rise to avian-specific digit loss. Dev. Dyn..

[bib32] Park K., Kang J., Subedi K.P., Ha J.-H., Park C. (2008). Canine polydactyl mutations with heterogeneous origin in the conserved intronic sequence of LMBR1. Genetics.

[bib33] Riddle R.D., Johnson R.L., Laufer E., Tabin C. (1993). Sonic hedgehog mediates the polarizing activity of the ZPA. Cell.

[bib34] Ristevski S., Tam P.P.L., Hertzog P.J., Kola I. (2002). Ets2 is expressed during morphogenesis of the somite and limb in the mouse embryo. Mech. Dev..

[bib35] Roelink H., Augsburger A., Heemskerk J., Korzh V., Norlin S., Ruiz i Altaba A., Tanabe Y., Placzek M., Edlund T., Jessell T.M. (1994). Floor plate and motor neuron induction by vhh-1, a vertebrate homolog of hedgehog expressed by the notochord. Cell.

[bib36] Sagai T., Hosoya M., Mizushina Y., Tamura M., Shiroishi T. (2005). Elimination of a long-range cis-regulatory module causes complete loss of limb-specific Shh expression and truncation of the mouse limb. Development.

[bib37] Sagai T., Amano T., Tamura M., Mizushina Y., Sumiyama K., Shiroishi T. (2009). A cluster of three long-range enhancers directs regional Shh expression in the epithelial linings. Development.

[bib38] Sanz-Ezquerro J.J., Tickle C. (2000). Autoregulation of Shh expression and Shh induction of cell death suggest a mechanism for modulating polarising activity during chick limb development. Development.

[bib39] Sarris E.G., Syrigos K.N., Saif M.W. (2013). Novel agents and future prospects in the treatment of pancreatic adenocarcinoma. J. Pancreas.

[bib40] Saunders J.W., Gasseling M., Fleischmayer R., Billingham R.E. (1968). Ectodermal–mesenchymal interactions in the origin of limb symmetry. Epithelial–Mesenchymal Interactions.

[bib41] Scherz P.J., McGlinn E., Nissim S., Tabin C.J. (2007). Extended exposure to Sonic hedgehog is required for patterning the posterior digits of the vertebrate limb. Dev. Biol..

[bib42] Smith J.C. (1980). The time required for positional signalling in the chick wing bud. J. Embryol. Exp. Morphol..

[bib43] Tanaka M., Cohn M.J., Ashby P., Davey M., Martin P., Tickle C. (2000). Distribution of polarizing activity and potential for limb formation in mouse and chick embryos and possible relationships to polydactyly. Development.

[bib44] Tickle C. (1981). The number of polarizing region cells required to specify additional digits in the developing chick wing. Nature.

[bib45] Tickle C., Barker H. (2012). 2012. The Sonic hedgehog gradient in the developing limb. Wiley Interdiscip. Rev.: Dev. Biol..

[bib46] Tickle C., Summerbell D., Wolpert L. (1975). Positional signalling and specification of digits in chick limb morphogenesis. Nature.

[bib47] Towers M., Mahood R., Yin Y., Tickle C. (2008). Integration of growth and specification in chick wing. Nature.

[bib48] Towers M., Signolet J., Sherman A., Sang H., Tickle C. (2011). Insights into bird wing evolution and digit specification from polarizing region fate maps. Nat. Commun..

[bib49] Verheyden J.M., Sun X. (2008). An Fgf/Gremlin inhibitory feedback loop triggers termination of limb bud outgrowth. Nature.

[bib50] Von Hoff D.D., LoRusso P.M., Rudin C.M., Reddy J.C., Yauch R.L., Tibes R., Weiss G.J., Borad M.J., Hann C.L., Brahmer J.R. (2009). Inhibition of the hedgehog pathway in advanced basal-cell carcinoma. N. Engl. J. Med..

[bib51] Wolpert L. (1969). Positional information and the spatial pattern of cellular differentiation. J. Theor. Biol..

[bib52] Wong F., Welten M.C.M., Anderson C., Bain A.A., Liu J., Wicks M.N., Pavlovska G., Davey M.G., Murphy P., Davidson D. (2013). eChickAtlas: an introduction to the database. Genesis.

[bib53] Yang Y., Drossopoulou G., Chuang P.T., Duprez D., Marti E., Bumcrot D., Vargesson N., Clarke J., Niswander L., McMahon A. (1997). Relationship between dose, distance and time in Sonic Hedgehog-mediated regulation of anteroposterior polarity in the chick limb. Development.

[bib54] Zeller R. (2010). The temporal dynamics of vertebrate limb development, teratogenesis and evolution. Curr. Opin. Genet. Dev..

[bib200] Zhao W., Dai F., Bonafede A., Schäfer S., Jung M., Yusuf F., Gamel A.J., Wang J., Brand-Saberi B. (2009). Histone deacetylase inhibitor, Trichostatin A, affects gene expression patterns during morphogensis of chicken limb buds in vivo. Cells Tissues Organs.

[bib56] Zhu J., Nakamura E., Nguyen M.-T., Bao X., Akiyama H., Mackem S. (2008). Uncoupling Sonic hedgehog control of pattern and expansion of the developing limb bud. Dev. Cell.

[bib57] Zuzarte-Luís V., Hurlé J.M. (2002). Programmed cell death in the developing limb. Int. J. Dev. Biol..

