# Supplementary material for: Direct functional consequences of ZRS enhancer mutation combine with secondary long range SHH signalling effects to cause preaxial polydactyly
Source: Dev Biol. 2014 Aug 15;392(2):209–20. doi: 10.1016/j.ydbio.2014.05.025 (PMC4111902; doi:10.1016/j.ydbio.2014.05.025)
Supplement: Supplementary file 4 — Supplementary Material [file mmc4.doc]

| **Family Information** | **Matrix** | **Leg Expression** | **Stage 25HH anterior leg** | **Leg ZPA expression** |
| --- | --- | --- | --- | --- |
| V$BRN5.04 | POU class 6 homeobox 1 (POU6F1) | NO |  |  |
| V$MYT1L.01 | Myelin transcription factor 1-like, neuronal C2HC zinc finger factor 1 | NO |  |  |
| V$HMX1.01 | H6 family homeobox 1 / NKX5-3 | NO |  |  |
|  |  |  |  |  |
| V$BRN5.04 | POU class 6 homeobox 1 (POU6F1) | NO |  |  |
| V$BARBIE.01 | Barbiturate-inducible element | NO |  |  |
| V$BRIGHT.01 | Bright, B cell regulator of IgH transcription | NO |  |  |
| V$HHEX.01 | Hematopoietically expressed homeobox, proline-rich homeodomain protein | NO |  |  |
| V$EN1.01 | Homeobox protein engrailed (en-1) |  |  | Repressor of SHH |
| V$PNR.01 | Photoreceptor-specific nuclear receptor subfamily 2, group E, member 3 (Nr2e3), DR1 sites | NO |  |  |
| V$BRN5.03 | Brn-5, POU-VI protein class (also known as emb and CNS-1) | NO |  |  |
| V$DLX3.01 | Distal-less 3 homeodomain transcription factor |  |  | NO |
| V$HOXB8.01 | Homeobox B8 / Hox-2delta |  | NO |  |
| V$HOXB9.02 | Homeobox B9 | NO |  |  |
| V$MSX.01 | Homeodomain proteins MSX-1 and MSX-2 |  | Too widely expressed |  |
| V$NKX61.01 | NK6 homeobox 1 | NO |  |  |
| V$PCE1.01 | Photoreceptor conserved element 1 | NO |  |  |
| V$GSH2.01 | Homeodomain transcription factor Gsh-2 | NO |  |  |
| V$HOXC8.01 | Homeobox C8 / Hox-3alpha |  |  | NO |
| V$NKX25.02 | Homeo domain factor Nkx-2.5/Csx, tinman homolog low affinity sites | NO |  |  |
| V$OCT1.05 | Octamer-binding factor 1 | Too widely expressed |  |  |
| V$S8.01 | Binding site for S8 type homeodomains |  | Too widely expressed |  |
| V$DLX2.01 | Distal-less homeobox 2 | NO |  |  |
